# Discussion on the fixed point problems with constraint inequalities

**DOI:** 10.1186/s13660-018-1818-4

**Published:** 2018-08-30

**Authors:** Badr Alqahtani, Rahmatollah Lashkaripour, Erdal Karapınar, Javad Hamzehnejadi

**Affiliations:** 1 Department of Mathematics, King Saud University, Riyadh, Saudi Arabia; 2 Department of Mathematics, University of Sistan and Baluchestan, Zahedan, Iran; 3 Department of Mathematics, Atilim University, Ankara, Turkey

**Keywords:** Comparable metric space, Fixed point, Generalized *α*-*h*-*ϕ*-contractions, Constraint inequalities

## Abstract

In this paper, we introduce the concept of comparable complete metric spaces and consider some fixed point theorems for mappings in the setting of incomplete metric spaces. We obtain the results of Ansari et al. [J. Fixed Point Theory Appl. 20:26, 2018] with weaker conditions. Moreover, we provide some corollaries and examples show that our main result is a generalization of existing results in the literature.

## Introduction and preliminaries

Let *Y* be a nonempty subset of a metric space $(X,d)$ and *T* be a function that map *Y* into itself. A fixed point of the mapping *T* is an element $x\in Y$ for which $Tx=x$. Fixed point theory plays a crucial role in nonlinear functional analysis and many authors have studied this notion. In 1922, Banach [7] reported the pioneer metric fixed point result for contraction mappings. Many authors have generalized this significant result in several directions; see e.g. [1–3, 8, 13].

Recently there have been many developments concerning the existence of fixed points for operators defined in a metric space equipped with a partial order. In 2016, Jleli and Samet [10] provided sufficient conditions for the existence of a fixed point of *T* satisfying the two constraint inequalities $Ax\preceq_{1} Bx$ and $Cx\preceq_{2} Dx$, where $T:X\rightarrow X$ defined on a complete metric space equipped with two partial orders ⪯_1_ and ⪯_2_ and $A,B,C,D:X\rightarrow X$ are self-map operators. In the other words, the problem is to investigate the existence a point $x\in X$ such that

1.1 $$ \textstyle\begin{cases} Tx=x;\\ Ax\preceq_{1} Bx ;\\ Cx\preceq_{2} Dx. \end{cases} $$

Before presenting the main result obtained in [10], let us recall some basic definitions and remarkable results introduced in [10] (see also e.g. [4, 5, 9, 15, 16]).

### Definition 1.1

Let $(X,d)$ be a metric space. A partial order “⪯” on *X* is *d*-regular if for any two sequences $\{u_{n}\}$ and $\{v_{n}\}$ in *X*, we have

$$ \lim_{n\rightarrow\infty}d(u_{n},u)=\lim_{n\rightarrow\infty}d(v_{n},v)=0, \qquad u_{n}\preceq v_{n}\quad\text{for all } n \quad \Longrightarrow\quad u\preceq v. $$

### Definition 1.2

Let $(X,\preceq)$ be an ordered set. A mapping $T:X\rightarrow X$ is said to be ⪯-preserving if $x\preceq y$ implies $T(x)\preceq T(y)$.

### Definition 1.3

Let “⪯_1_” and “⪯_2_” be two partial orders on *X* and operators $T,A,B,C,D:X \rightarrow X$ be given. The operator *T* is called $(A,B,C,D,\preceq_{1},\preceq _{2})$-stable if for all $x\in X$,

$$Ax \preceq_{1} Bx \quad \Longrightarrow\quad CTx \preceq_{2} DTx. $$

### Example 1.4

Let $X=\mathbb{ R}$ and consider the standard order “≤” on *X*. Let $A,B,C,D:X\rightarrow X$ be the operators defined by

$$ \begin{gathered} Ax = x, \qquad Bx = x^{2}, \qquad Cx = \exp(x), \\ Dx = \exp\bigl(x^{2}-2x+2\bigr), \qquad Tx = x + 1, \quad x\in \mathbb{R}. \end{gathered} $$

Then the operator *T* is $(A,B,C,D,\leq,\leq)$-stable.

Let us denote by Ψ the set of all lower semi-continuous functions $\psi:[0,\infty)\rightarrow[0,\infty)$ such that $\psi^{-1}\{0\}=\{0\}$. The main theorem presented in [10] is given by the following result.

### Theorem 1.5

*Let* $(X,d)$ *be a complete metric space endowed with two partial orders “*⪯_1_*” and “*⪯_2_*”*. *Let operators* $T,A,B,C,D:X\rightarrow X$ *be given*. *Suppose that the following conditions are satisfied*:

(i) $\preceq_{i}$ *is* *d*-*regular*, $i = 1,2$;
(ii) *A*, *B*, *C* *and* *D* *are continuous*;
(iii) *there exists* $x_{0} \in X$ *such that* $Ax_{0} \preceq_{1} Bx_{0}$;
(iv) *T* *is* $(A,B,C,D,\preceq_{1},\preceq_{2})$-*stable*;
(v) *T* *is* $(C,D,A,B,\preceq_{2},\preceq_{1})$-*stable*;
(vi) *there exists* $\psi\in\Psi$ *such that*
  $$ (Ax\preceq_{1} Bx\textit{ and } Cy\preceq_{2} Dx) \quad \Longrightarrow\quad d(Tx,Ty)\leq d(x,y)-\psi\bigl(d(x,y)\bigr). $$

*Then the sequence* $\{T^{n}x_{0}\}$ *converges to some* $x^{*}\in X$ *which is a solution to* (1.1).

Ansari *et al.* in [6] proved that $x^{*}$ is the unique solution to (1.1) and removed the continuity of *C* and *D*.

In our main theorem, we replace the completeness assumption of the space *X* with weaker conditions. Also we consider a more general condition in assumption (vi). For this purpose, we review the concept of generalized *α*-*h*-*ϕ*-contraction type mapping and some examples introduced in [14]. Also, we introduce new concepts to remove the completeness assumption of the space *X*.

### Definition 1.6

([11])

Let $T:X \rightarrow X$ be a mapping and $\alpha:X\times X\rightarrow [0,\infty)$ be a function. The mapping *T* is said to be *α*-admissible if

$$\alpha(x,y) \geq1\quad\text{implies}\quad \alpha(Tx, Ty) \geq1. $$

An *α*-admissible mapping *T* is said to be triangular *α*-admissible [12] if

$$\alpha(x,y)\geq1\quad\text{and}\quad\alpha(y,z)\geq1\quad \text{imply}\quad \alpha(x,z)\geq1. $$

### Lemma 1.7

([11])

*Let* $T:X\rightarrow X$ *be a triangular* *α*-*admissible map*. *Assume that there exists* $x_{0}\in X$ *such that* $\alpha(x_{0},Tx_{0})\geq1$. *Define a sequence* $\{x_{n}\}$ *by* $x_{n+1} = Tx_{n}$. *Then*, *we have* $\alpha(x_{n},x_{m})\geq1$ *for all* $m, n \in\mathbb{N}$ *with* $n < m$.

### Definition 1.8

([11])

Let $(X,d)$ be a metric space and $\alpha: X\times X\rightarrow[0,\infty )$ be a function. A sequence $\{x_{n}\}$ is said to be *α*-regular if the following condition is satisfied:

If $\{x_{n}\}$ is a sequence in *X* such that $\alpha(x_{n}, x_{n+1}) \geq 1$ for all $n\in\mathbb{N}$ and $x_{n}\rightarrow x\in X$ as $n\rightarrow\infty$, then there exists a subsequence $\{x_{n_{k}}\}$ of $\{x_{n}\}$ such that $\alpha(x_{n_{k}}, x)\geq1$ for all *k*.

Recently we introduced a new class of mappings which contain a Geraghty-contraction type mapping and some of its extensions and some of weakly contractive type mappings as a subclass.

### Definition 1.9

([14])

Let $(X,d)$ be a metric space. Define $\mathcal{H}(X)$ by the class of all mappings $h: X\times X\rightarrow[0,1)$ which satisfies the following condition:

$$\lim_{n\rightarrow\infty}h(x_{n},y_{n})=1 \quad \Longrightarrow\quad\lim_{n\rightarrow\infty}d(x_{n},y_{n})=0, $$

for all sequences $\{x_{n}\}$ and $\{y_{n}\}$ in *X* such that the sequence $\{d(x_{n},y_{n})\}$ is decreasing and convergent.

### Example 1.10

([14])

Let $h:\mathbb{R}\times\mathbb{R}\rightarrow[0,1)$, defined by

(i) $h_{1}(x,y)=\frac{t}{t+x^{2}+y^{2}}$, for some $t\in[0,\infty)$.
(ii) $h_{2}(x,y)=k$, for some $k\in(0,1)$.

Then $h_{1},h_{2}\in\mathcal{H}(\mathbb{R})$.

Let $\mathcal{F}$ be the class of those functions $\beta: [0,\infty )\rightarrow[0, 1)$ satisfying the following condition:

$$\beta(t_{n})\rightarrow1\quad\text{implies} \quad t_{n} \rightarrow0. $$

### Example 1.11

([14])

Let $(X,d)$ be a metric space and $\beta\in\mathcal{F}$. Define $h_{1},h_{2}:X\times X\rightarrow[0,1)$, by

$$ \begin{gathered} h_{1}(x,y)=\beta\bigl(d(x,y)\bigr); \\ h_{2}(x,y)=\beta\bigl(M_{a}(x,y)\bigr), \end{gathered} $$

where $\beta\in\mathcal{F}$ and for all $x,y\in X$

$$M_{a}(x,y)=\max \biggl\{ d(x,y),d(x,Tx),d(y,Ty),\frac{d(x,Ty)+d(y,Tx)}{2} \biggr\} . $$

Then, $h_{1},h_{2}\in\mathcal{H}$.

### Definition 1.12

([14])

Let $(X,d)$ be a metric space and $\alpha:X\times X\rightarrow\mathbb {R}$ be a function. A mapping $T:X\rightarrow X$ is said to be generalized *α*-*h*-*ϕ*-contraction if there exist $h\in \mathcal{H}(X)$ and $\phi\in\Phi$ such that

$$ \alpha(x,y)\phi\bigl(d(Tx,Ty)\bigr)\leq h(x,y)\phi \bigl(M_{a}(x,y)\bigr). $$

One of extensions of the Banach contraction principle that extend, generalize, and improve some existing results, was given by Lashkaripour et al. as follows.

### Theorem 1.13

([14])

*Let* $(X,d)$ *be a complete metric space*, $\alpha:X\times X\rightarrow \mathbb{R}$ *be a function and* $T : X \rightarrow X$ *be a mapping*. *Suppose that the following conditions are satisfied*:

(i) *T* *is a generalized* *α*-*h*-*ϕ*-*contraction type mapping*;
(ii) *T* *is triangular* *α*-*admissible*;
(iii) *there exists* $x_{1} \in X$ *such that* $\alpha(x_{1},Tx_{1}) \geq1$;
(iv) *T* *is continuous or for all sequences* $\{x_{n}\},\{y_{n}\} \subseteq X$ *that* $\alpha(x_{n},y_{n})\neq0$, $\forall n\in\mathbb{N}$, *the following condition is satisfied*:
  $$\lim_{n\rightarrow\infty}h(x_{n},y_{n})=1 \quad \Longrightarrow\quad \lim_{n\rightarrow \infty}d(Tx_{n},Ty_{n})=0. $$

*Then* *T* *has a fixed point* $x^{*}\in X$, *and* $\{T^{n}x_{1}\}$ *converges to* $x^{*}$.

Next, we introduce the concept of comparable sequences and comparable complete metric spaces.

### Definition 1.14

Let $(X,\preceq)$ be an ordered space. A sequence $\{x_{n}\}$ is called a comparable sequence, if

$$(\forall n,k; x_{n}\preceq x_{n+k})\quad \mbox{or}\quad (\forall n,k; x_{n+k}\preceq x_{n}). $$

### Example 1.15

Let $X=\mathbb{ R}$ and consider the standard order “≤” on *X*. Then every monotone sequence is comparable sequence.

### Definition 1.16

Let $(X,\preceq,d)$ be an ordered metric space. *X* is said to be comparable complete if every Cauchy comparable sequence is convergent.

It is easy to see that every complete metric space is comparable complete and that the converse is not true. In the next example, *X* is comparable complete but it is not complete.

### Example 1.17

Let $X=\mathbb{Q}$. Suppose that

$$x\preceq y \quad \Longleftrightarrow\quad \exists k,s\in\mathbb{N}\cup\{0\}: |x-y|=10^{-k}\bigl(1-10^{-s}\bigr). $$

Clearly, $\mathbb{Q}$ with the Euclidean metric is not a complete metric space, but it is comparable complete metric space. If $\{x_{n}\}$ is an arbitrary Cauchy comparable sequence in *X*, then the sequence is convergent in $\mathbb{R}$. We prove that *x* is a rational number. In the contrary case let $x\in\mathbb{Q}^{c}$. Since $\{x_{n}\}$ is a comparable sequence, for all $m,n\in\mathbb{N}$ there exist $k,s\in \mathbb{N}\cup\{0\}$ such that

$$|x_{n}-x_{m}|=10^{-k}\bigl(1-10^{-s} \bigr). $$

Suppose that $m\rightarrow\infty$, then there exists $r\in\mathbb {Q}$ such that $|x_{n}-x|=10^{-r}$, which is a contradiction. Therefore the space $\mathbb{Q}$ with this order is a comparable complete metric space. Note that for all $x\in\mathbb{Q}$ there exists a comparable sequence $\{x_{n}\}\subseteq\mathbb{Q}$ such that $\lim_{n\rightarrow \infty}x_{n}=x$.

### Definition 1.18

Let $(X,\preceq,d)$ be an ordered metric space. A mapping $f:X\rightarrow X$ is comparable continuous in $a\in X$ if for each comparable sequence $\{a_{n}\}$ in *X* if $a_{n}\rightarrow a$, then $f(a_{n})\rightarrow f(a)$. Also, *f* is comparable continuous on X if *f* is comparable continuous in each $a\in X$.

Every continuous function is a comparable continuous function, but the converse is not true in general.

### Example 1.19

Let $X=\mathbb{R}$ with the Euclidean metric and usual order “≤”. Let $f:\mathbb{R}\rightarrow\mathbb{R}$ defined by $f(x)=[x]$. The function *f* is not a continuous function. Define the relation “⪯” on $\mathbb{R}$ as follows:

$$x\preceq y\quad \Longleftrightarrow\quad x\geq y. $$

It is easy to see that the function *f* is a comparable continuous function.

### Definition 1.20

Let $(X,\preceq)$ be an ordered space and $T:X\rightarrow X$ be a mapping. $x_{0}\in X$ is said to be *T*-comparable if for all $n\in\mathbb{N}$, $x_{0}$ and $T^{n}x_{0}$ be comparable and define

$$ \mathcal{J_{T}}=\bigl\{ x_{0}\in X; \bigl(\forall n\in \mathbb{N}: x_{0}\preceq T^{n}x_{0} \bigr)\text{ or } \bigl(\forall n\in\mathbb{N}; T^{n}x_{0}\preceq x_{0}\bigr)\bigr\} . $$

### Example 1.21

Let $X=\mathbb{R}$ with the Euclidean metric and usual order “≤”. If define $T:X\rightarrow X$ by $T(x)=x^{2} $, then $\mathcal{J_{T}}=\mathbb{R}$. Also if

$$ g(x)= \textstyle\begin{cases} -1 & x\geq0,\\ 1& x< 0, \end{cases} $$

then $\mathcal{J_{g}}=\emptyset$.

### Proposition 1.22

*Let* $(X,\preceq)$ *be an ordered set and* $T: X \rightarrow X$ *be* ⪯-*preserving*. *Let* $\{x_{n}\}$ *be Picard iterative sequence with initial point* $x_{0}\in\mathcal{J_{T}}$, *i*.*e*. $x_{n} = T^{n}(x_{0})$. *Then* $\{x_{n}\}$ *is a comparable sequence*.

### Proof

Let $n,k\in\mathbb{N}$ and for all $k\in\mathbb{N}$, $x_{0}\preceq T^{k}x_{0}=x_{k}$. Since *T* is ⪯-preserving, $x_{1}=Tx_{0}\preceq Tx_{n}=x_{k+1}$. Inductively for all $n\in\mathbb{N}$ we can prove that $x_{n}\preceq x_{n+k}$. □

## Main result

Let Φ be the family of functions $\phi: [0,\infty)\rightarrow [0,\infty)$ satisfying the following conditions:

1. *ϕ* is continuous and non-decreasing;
2. $\phi(t)=0$ if and only if $t=0$.

In the following theorem, which is our first main result, we weaken assumption (ii) and (vi) of Theorem 1.5. Moreover, we remove the completeness assumption of the space in Theorem 1.5.

### Theorem 2.1

*Let* $(X,d,\preceq)$ *be a comparable complete metric space*(*not necessarily complete*). *Let* ⪯_1_ *and* ⪯_2_ *be two partial order over* *X*. *Also*, *let operators* $T,A,B,C,D:X \rightarrow X $ *be given*. *Suppose that the following conditions are satisfied*:

(i) $\preceq_{i}$ *is* *d*-*regular*, $i = 1,2$ *and* *T* *is* ⪯-*preserving and triangular* *α*-*admissible*;
(ii) *A*, *B* *and* *T* *are comparable continuous*;
(iii) *there exists* $x_{0} \in\mathcal{J_{T}}$ *such that* $Ax_{0} \preceq_{1} Bx_{0}$ *and* $\alpha(x_{0},Tx_{0})\geq1$;
(iv) *T* *is* $(A,B,C,D,\preceq_{1},\preceq_{2})$-*stable*;
(v) *T* *is* $(C,D,A,B,\preceq_{2},\preceq_{1})$-*stable*;
(vi) *there exist* $h\in\mathcal{H}(X)$ *and* $\phi\in\Phi$ *such that*
  $$ (Ax\preceq_{1} Bx\textit{ and }Cy\preceq_{2} Dy) \quad \Longrightarrow\quad \alpha (x,y)\phi\bigl(d(Tx,Ty)\bigr)\leq h(x,y)\phi \bigl(M_{a}(x,y)\bigr). $$

*Then the sequence* $T^{n}x_{0}$ *converges to some* $x^{*}\in X$ *which is a solution to* (1).

### Proof

From condition (iii), there exists $x_{0} \in\mathcal{J_{T}}$ such that

$$Ax_{0} \preceq_{1} Bx_{0} \quad\text{and}\quad \alpha(x_{0},Tx_{0})\geq1. $$

Define the sequence $\{x_{n}\}$ by $x_{n}=Tx_{n-1}$, for all $n\in\mathbb {N}$. Applying Proposition 1.22, $\{x_{n}\}$ is a comparable sequence. If $x_{n_{0}}=x_{n_{0}+1}$ for some $n_{0}\in\mathbb{N}$, then $Tx_{n_{0}}=x_{n_{0}+1}=x_{n_{0}}$, and hence the proof is completed. Now, let $x_{n}\neq x_{n+1}$, $n=0,1,2,\ldots $ . Since $Ax_{0}\preceq_{1} Bx_{0}$ and *T* is $(A,B,C,D,\preceq_{1},\preceq_{2})$-stable, we have

$$Ax_{0} \preceq_{1} Bx_{0} \quad \Longrightarrow\quad CTx_{0} \preceq_{2} DTx_{0}, $$

that is, $Cx_{1} \preceq_{2} Dx_{1}$. Hence

$$Ax_{0} \preceq_{1} Bx_{0}\quad \text{and}\quad Cx_{1} \preceq_{2} Dx_{1}. $$

Continuing this process, by induction, for all $n\in\mathbb{N}$ we get

2.1 $$ Ax_{2n}\preceq_{1} Bx_{2n}\quad \text{and}\quad Cx_{2n+1}\preceq_{2} Dx_{2n+1}. $$

Also, applying Lemma 1.7 for all $m, n \in\mathbb{N}$ with $n < m$, we have

2.2 $$ \alpha(x_{n},x_{m})\geq1. $$

Since $\{x_{n}\}$ is comparable, applying (2.1), (2.2) and (vi), by symmetry, for $n=1,2,\ldots$ , we have

2.3 $$\begin{aligned} \phi\bigl(d(x_{n},x_{n+1})\bigr) \leq& \alpha(x_{n-1},x_{n})\phi \bigl(d(x_{n},x_{n+1}) \bigr) \\ =&\alpha(x_{n-1},x_{n})\phi\bigl(d(Tx_{n-1},Tx_{n}) \bigr) \\ \leq&h(x_{n-1},x_{n})\phi\bigl(M_{a}(x_{n-1},x_{n}) \bigr) \\ < & \phi\bigl(M_{a}(x_{n-1},x_{n})\bigr). \end{aligned}$$

Also, we have

$$\begin{aligned} M_{a}(x_{n-1},x_{n}) =&\max \biggl\{ d(x_{n-1},x_{n}),d(x_{n-1},Tx_{n-1}),d(x_{n},Tx_{n}), \frac {d(x_{n-1},Tx_{n})+d(x_{n},Tx_{n-1})}{2} \biggr\} \\ =&\max \biggl\{ d(x_{n-1},x_{n}),d(x_{n-1},x_{n}),d(x_{n},x_{n+1}), \frac {d(x_{n-1},x_{n+1})+d(x_{n},x_{n})}{2} \biggr\} \\ =&\max \biggl\{ d(x_{n-1},x_{n}),d(x_{n},x_{n+1}), \frac {d(x_{n-1},x_{n+1})}{2} \biggr\} \\ \leq& \max \biggl\{ d(x_{n-1},x_{n}),d(x_{n},x_{n+1}), \frac {d(x_{n-1},x_{n})+d(x_{n},x_{n+1})}{2} \biggr\} \\ =& \max\bigl\{ d(x_{n-1},x_{n}),d(x_{n},x_{n+1}) \bigr\} . \end{aligned}$$

If $M_{a}(x_{n-1},x_{n})=d(x_{n},x_{n+1})$, applying (2.3), we deduce that

$$\begin{aligned} \phi\bigl(d(x_{n},x_{n+1})\bigr) < & \phi \bigl(M_{a}(x_{n-1},x_{n})\bigr) \\ =&\phi\bigl(d(x_{n},x_{n+1})\bigr), \end{aligned}$$

which is a contradiction. Thus, we conclude that

2.4 $$ M_{a}(x_{n-1},x_{n})=d(x_{n-1},x_{n}), \quad\forall n\in\mathbb{N}. $$

Now, from (2.3) and (2.4), we get

$$\phi\bigl(d(x_{n},x_{n+1})\bigr)< \phi\bigl(d(x_{n-1},x_{n}) \bigr), \quad \forall n\in \mathbb{N}. $$

The monotony of *ϕ* implies that

$$d(x_{n},x_{n+1})< d(x_{n-1},x_{n}), \quad \forall n\in\mathbb{N}. $$

We deduce that the sequence $\{d(x_{n}, x_{n+1})\}$ is nonnegative and decreasing. Consequently, there exists $r \geq0$ such that $\lim_{n\rightarrow\infty }d(x_{n},x_{n+1})=r$. We prove that $r = 0$. In the contrary case, suppose that $r > 0$. Then from (2.3) and (2.4), we have

$$0< \frac{\phi(d(x_{n},x_{n+1}))}{\phi(d(x_{n-1},x_{n}))}\leq h(x_{n-1},x_{n}), $$

which implies that $\lim_{n\rightarrow\infty}h(x_{n-1},x_{n})=1$. Since $h \in\mathcal{H}$,

$$\lim_{n\rightarrow\infty}d(x_{n-1},x_{n})=0. $$

This implies that $r=0$, which is a contradiction. Therefore

$$\lim_{n\rightarrow\infty}d(x_{n},x_{n+1})=0. $$

Now, we shall prove that $\{x_{n}\}$ is a Cauchy sequence in comparable complete metric space $(X,\preceq,d)$. Suppose, on the contrary, that $\{x_{n}\}$ is not a Cauchy sequence. Thus, there exists $\epsilon>0$ such that, for all $k\in\mathbb{N}$, there exist $n_{k}> m_{k}>k$ such that

$$d(x_{m_{k}},x_{n_{k}})\geq\epsilon. $$

Also, choosing $m_{k}$ as small as possible, it may be assumed that

$$d(x_{m_{k}},x_{n_{k}-1})< \epsilon. $$

Hence for each $k\in\mathbb{N}$, we have

$$\begin{aligned} \epsilon \leq& d(x_{m_{k}},x_{n_{k}})\leq d(x_{m_{k}},x_{n_{k}-1})+d(x_{n_{k}-1},x_{n_{k}}) \\ \leq& \epsilon+d(x_{n_{k}-1},x_{n_{k}}). \end{aligned}$$

Letting $k\rightarrow\infty$ in the above inequality, we get

$$\lim_{n\rightarrow\infty} d(x_{n_{k}},x_{m_{k}})=\epsilon. $$

The triangle inequality implies that

2.5 $$ \lim_{n\rightarrow\infty} d(x_{n_{k}+1},x_{m_{k}})= \epsilon,\qquad \lim_{n\rightarrow\infty} d(x_{n_{k}},x_{m_{k}-1})= \epsilon,\qquad \lim_{n\rightarrow\infty} d(x_{n_{k}+1},x_{m_{k}+1})= \epsilon. $$

We see that, for all $k\in\mathbb{N}$, there exists $i_{k}\in\{0,1\}$ such that

$$n_{k} - m_{k} + i_{k} \equiv1(2). $$

Now, applying (2.1), for all $k > 1$, we deduce that

$$Ax_{n_{k}} \preceq_{1} Bx_{n_{k}} \quad\text{and}\quad Cx_{m_{k}-i_{k}}\preceq_{2} Dx_{m_{k}-i_{k}}, $$

or

$$Ax_{m_{k}-i_{k}} \preceq_{1} Bx_{m_{k}-i_{k}} \quad\text{and}\quad Cx_{n_{k}}\preceq_{2} Dx_{n_{k}}. $$

Now, applying (vi), for $k \in\mathbb{N}$, we conclude that

2.6 $$ \begin{aligned}[b] \phi\bigl(d(x_{n_{k}+1},x_{m_{k}-i_{k}+1})\bigr)&\leq \alpha(x_{n_{k}},x_{m_{k}-i_{k}})\phi \bigl(d(x_{n_{k}+1},x_{m_{k}-i_{k}+1}) \bigr) \\ &=\alpha(x_{n_{k}},x_{m_{k}-i_{k}})\phi\bigl(d(Tx_{n_{k}},Tx_{m_{k}-i_{k}}) \bigr) \\ & \leq h(x_{n_{k}},x_{m_{k}-i_{k}})\phi\bigl(M_{a}(x_{n_{k}},x_{m_{k}-i_{k}}) \bigr). \end{aligned} $$

Also, for any $k\in\mathbb{N}$, we have

$$\begin{aligned} M_{a}(x_{n_{k}},x_{m_{k}-i_{k}}) = & \max \biggl\{ d(x_{n_{k}},x_{m_{k}-i_{k}}),d(x_{n_{k}},Tx_{n_{k}}),d(x_{m_{k}-i_{k}},Tx_{m_{k}-i_{k}}),\\ & \frac {d(x_{n_{k}},Tx_{m_{k}-i_{k}})+d(x_{m_{k}-i_{k}},Tx_{n_{k}})}{2} \biggr\} \\ = & \max \biggl\{ d(x_{k},x_{m_{k}-i_{k}}),d(x_{n_{k}},x_{n_{k}+1}),d(x_{m_{k}-i_{k}},x_{m_{k}-i_{k}+1}),\\ & \frac {d(x_{n_{k}},x_{m_{k}-i_{k}+1})+d(x_{m_{k}-i_{k}},x_{n_{k}+1})}{2} \biggr\} \\ \leq& \max \biggl\{ d(x_{n_{k}},x_{m_{k}-i_{k}}),d(x_{n_{k}},x_{n_{k}+1}),d(x_{m_{k}-i_{k}},x_{m_{k}-i_{k}+1}),\\ & \frac {d(x_{n_{k}},x_{m_{k}-i_{k}})+d(x_{m_{k}-i_{k}},x_{m_{k}-i_{k}+1})}{2} + \frac{d(x_{m_{k}-i_{k}},x_{n_{k}})+d(x_{n_{k}},x_{n_{k}+1})}{2} \biggr\} . \end{aligned}$$

Since $\lim_{k\rightarrow\infty}d(x_{n_{k}},x_{n_{k}+1})=0$,

2.7 $$ \lim_{k\rightarrow\infty}M_{a}(x_{n_{k}},x_{m_{k}-i_{k}})= \lim_{k\rightarrow \infty}d(x_{n_{k}},x_{m_{k}-i_{k}}). $$

Combining (2.6) and (2.7) with the continuity of *ϕ*, we get

$$\lim_{k\rightarrow\infty}\phi\bigl(d(x_{n_{k}+1},x_{m_{k}-i_{k}+1})\bigr) \leq\lim_{k\rightarrow\infty}h(x_{n_{k}},x_{m_{k}-i_{k}})\lim _{k\rightarrow\infty }\phi\bigl(d(x_{n_{k}},x_{m_{k}-i_{k}})\bigr). $$

Applying (2.5), we deduce that

$$\lim_{k\rightarrow\infty}h(x_{n_{k}},x_{m_{k}-i_{k}})=1. $$

Since $h\in\mathcal{H}(X)$,

$$\lim_{k\rightarrow\infty}d(x_{n_{k}},x_{m_{k}-i_{k}})=0, $$

which is a contradiction. Thus, $\{x_{n}\}$ is Cauchy comparable and so there exists $x^{*}\in X$ such that $\lim_{n\rightarrow\infty} x_{n}=x^{*}$. Since *T* is a comparable continuous function,

$$\lim_{n\rightarrow\infty} x_{n+1}=\lim_{n\rightarrow\infty}Tx_{n}=Tx^{*}. $$

Therefore

2.8 $$ Tx^{*}=x^{*}. $$

*A* and *B* are comparable continuous and $\{x_{2n}\}$ is a comparable sequence, therefore

$$\lim_{n\rightarrow\infty}d\bigl(Ax_{2n},Ax^{*}\bigr)=\lim _{n\rightarrow\infty }d(Bx_{2n},Bx_{2n})=0. $$

Since ⪯_1_ is *d*-regular, (2.1) implies that

2.9 $$ Ax^{*}\preceq_{1} Bx^{*}. $$

Since *T* is $(A,B,C,D,\preceq_{1},\preceq_{2})$-stable, applying (2.9), we have

$$CTx^{*}\preceq_{2} DTx^{*}. $$

This implies that

2.10 $$ Cx^{*}\preceq_{2} Dx^{*}. $$

Applying (2.8), (2.9) and (2.10), we deduce that $x^{*}$ is a solution of (2.1). □

In the following theorem, we omit the continuity condition of the mapping *T* in Theorem 2.1.

### Theorem 2.2

*Let* $(X,d,\preceq)$ *be a comparable complete metric space*(*not necessarily complete*). *Let* ⪯_1_ *and* ⪯_2_ *be two partial order over* *X*. *Also*, *let operators* $T,A,B,C,D:X \rightarrow X $ *be given*. *Suppose that the following conditions are satisfied*:

(i) $\preceq_{i}$ *is* *d*-*regular*, $i = 1,2$ *and* *T* *is* ⪯-*preserving and triangular* *α*-*admissible*;
(ii) *A*, *B* *are comparable continuous*;
(iii) *there exists* $x_{0} \in\mathcal{J_{T}}$ *such that* $Ax_{0} \preceq_{1} Bx_{0}$ *and* $\alpha(x_{0},Tx_{0})\geq0$;
(iv) *the sequence* $\{T^{2n}x_{0}\}$ *is* *α*-*regular*;
(v) *T* *is* $(A,B,C,D,\preceq_{1},\preceq_{2})$-*stable and* $(C,D,A,B,\preceq_{2},\preceq_{1})$-*stable*;
(vi) *there exist* $h\in\mathcal{H}(X)$ *and* $\phi\in\Phi$ *such that for all sequences* $\{x_{n}\},\{y_{n}\}\subseteq X$ *where* $\alpha (x_{n},y_{n})\neq0$, $\forall n\in\mathbb{N}$, *the following conditions are satisfied*:
  $$\begin{aligned}& \lim_{n\rightarrow\infty}h(x_{n},y_{n})=1\quad \Longrightarrow\quad \lim_{n\rightarrow \infty}d(Tx_{n},Ty_{n})=0; \\& (Ax\preceq_{1} Bx \textit{ and } Cy\preceq_{2} Dy) \quad \Longrightarrow\quad \alpha (x,y)\phi\bigl(d(Tx,Ty)\bigr)\leq h(x,y)\phi \bigl(M_{a}(x,y)\bigr). \end{aligned}$$

*Then* *T* *has a fixed point* $x^{*}\in X$, *and* $\{T^{n}x_{0}\}$ *converges to* $x^{*}$.

### Proof

From condition (iii), there exists $x_{0} \in\mathcal{J_{T}}$ such that

$$Ax_{0} \preceq_{1} Bx_{0}\quad \text{and}\quad \alpha(x_{0},Tx_{0})\geq1. $$

Define the sequence $\{x_{n}\}$ by $x_{n}=Tx_{n-1}$, for all $n\in\mathbb{N}$. Following the proof of Theorem 2.1, we know that, for $n=0,1,\ldots$ ,

2.11 $$ Ax_{2n}\preceq_{1} Bx_{2n},\qquad Cx_{2n+1}\preceq_{2} Dx_{2n+1}\quad \text{and}\quad \alpha(x_{n},x_{n+1})\geq1 , $$

and the sequence $\{x_{n}\}$ is convergent to some $x^{*}\in X$. Also, we have

2.12 $$ Ax^{*}\preceq_{1} Bx^{*}. $$

Now, we prove that $Tx^{*}=x^{*}$. In the contrary case suppose that $Tx^{*}\neq x^{*}$. Since the sequence $\{x_{2n}\}$ is *α*-regular, there exists a subsequence $\{x_{2n_{k}}\}$ such that $\alpha(x_{2n_{k}},x^{*})\geq1$ for all $k\in\mathbb{N}$. Without loss of generality, we assume that

2.13 $$ \alpha\bigl(x_{2n}, x^{*}\bigr)\geq1,\quad n=0,1,2,\ldots. $$

Applying (2.11), (2.13), for $n=0,1,\ldots$ , we get

2.14 $$\begin{aligned} \phi\bigl(d\bigl(x_{2n+1},Tx^{*}\bigr)\bigr) =&\phi\bigl(d \bigl(Tx_{2n},Tx^{*}\bigr)\bigr) \\ \leq&\alpha\bigl(x_{2n},x^{*}\bigr)\phi\bigl(d\bigl(Tx_{2n},Tx^{*} \bigr)\bigr) \\ \leq&h\bigl(x_{2n},x^{*}\bigr)\phi\bigl(M_{a} \bigl(x_{2n},x^{*}\bigr)\bigr). \end{aligned}$$

Also, we have

$$\begin{aligned} M_{a}\bigl(x_{2n},x^{*}\bigr) =&\max \biggl\{ d \bigl(x_{2n},x^{*}\bigr),d(x_{2n},Tx_{2n}),d \bigl(x^{*},Tx^{*}\bigr),\frac {d(x_{2n},Tx^{*})+d(x^{*},Tx_{2n})}{2} \biggr\} \\ =&\max \biggl\{ d\bigl(x_{2n},x^{*}\bigr),d(x_{2n},x_{2n+1}),d \bigl(x^{*},Tx^{*}\bigr),\frac {d(x_{2n},Tx^{*})+d(x^{*},x_{2n+1})}{2} \biggr\} . \end{aligned}$$

Since $\lim_{n\rightarrow\infty}d(x_{2n},x^{*})=0$, $\lim_{n\rightarrow \infty}M_{a}(x_{2n},x^{*})=d(x^{*},Tx^{*})$. Applying (2.14) and the continuity of *ϕ*, we get $\lim_{n\rightarrow\infty}h(x_{2n},x^{*})=1$, and so

$$d\bigl(x^{*},Tx^{*}\bigr)=\lim_{n\rightarrow\infty}d\bigl(Tx_{2n},Tx^{*} \bigr)=0. $$

This is a contradiction. Therefore $Tx^{*}=x^{*}$. Since *T* is $(A,B,C,D,\preceq_{1},\preceq_{2})$-stable, applying (2.12), we have

$$ CTx^{*}\preceq_{2} DTx^{*}. $$

Therefore, $Cx^{*}\preceq_{2} Dx^{*}$. This implies that $x^{*}$ is a solution of (1.1). □

For the uniqueness of the solution of (1.1) we will consider the following condition.

$(H1)$
For all $x,y\in \operatorname{Fix}(T)$, there exists $z\in X$ such that $\alpha(x, z)\geq1$ and $\alpha(z,y)\geq1$.

### Theorem 2.3

*Adding condition* $(H1)$ *to the hypotheses of Theorem *2.1 (*resp*. *Theorem *2.2), *we see that* $x^{*}$ *is the unique fixed point of* *T*.

### Proof

Let $y^{*}\in X$ be another solution of (1.1), that is,

2.15 $$ Ty^{*}=y^{*},\qquad Ay^{*}\preceq_{1} By^{*},\qquad Cy^{*}\preceq_{2} Dy^{*}. $$

we show that $x^{*}=y^{*}$. In the contrary case, let $x^{*}\neq y^{*}$. There exists $z\in X$ such that

$$\alpha\bigl(x^{*},z\bigr)\geq1\quad \text{and}\quad\alpha\bigl(z,y^{*}\bigr)\geq1. $$

Since *T* is triangular *α*-admissible, we have $\alpha (x^{*},y^{*})\geq1$. Now, $Ax^{*}\preceq_{1} Bx^{*}$ and $Cy^{*}\preceq_{2}Dy^{*} $, which implies that

2.16 $$\begin{aligned} \phi\bigl(d\bigl(x^{*},y^{*}\bigr)\bigr) =&\phi\bigl(d\bigl(Tx^{*},Ty^{*} \bigr)\bigr) \\ \leq&\alpha\bigl(x^{*},y^{*}\bigr)\phi\bigl(d\bigl(Tx^{*},Ty^{*}\bigr)\bigr) \\ \leq&h\bigl(x^{*},y^{*}\bigr)\phi\bigl(M_{a}\bigl(x^{*},y^{*}\bigr)\bigr) \\ < &\phi\bigl(M_{a}\bigl(x^{*},y^{*}\bigr)\bigr). \end{aligned}$$

On the other hand, we have

2.17 $$\begin{aligned} M_{a}\bigl(x^{*},y^{*}\bigr) =&\max \biggl\{ d\bigl(x^{*},y^{*} \bigr),d\bigl(x^{*},Tx^{*}\bigr),d\bigl(y^{*},Ty^{*}\bigr),\frac {d(x^{*},Ty^{*})+d(y^{*},Tx^{*})}{2} \biggr\} \\ =& d\bigl(x^{*},y^{*}\bigr). \end{aligned}$$

Applying (2.16) and (2.17), we have $\phi(d(x^{*},y^{*}))<\phi (d(x^{*},y^{*}))$, which is a contradiction. This implies that $x^{*}=y^{*}$, and so the fixed point of *T* is unique. □

### Example 2.4

Let $X=[-2,3)$ and define relation “⪯” on $\mathbb{R}$ as follows:

$$x\preceq y\quad \Longleftrightarrow\quad [x]=[y]\quad \text{and}\quad x\geq y. $$

The space *X* with the Euclidean metric is not a complete metric space, but it is comparable complete metric space. We take ${\preceq_{1}}={\preceq _{2}}={\leq}$. Let $T:X\rightarrow X$ be the mapping defined by

$$T(x)=\frac{1}{2}\bigl(x-[x]\bigr), \quad\forall x\in X. $$

For all $x,y \in X$ such that $x\preceq y$, we have $Tx\preceq Ty$. Therefore *T* is ⪯-preserving. consider the mappings $A,B,C,D:X\rightarrow X$ defined by $D(x)=-4x+1$,

$$ \begin{gathered} A(x)= \textstyle\begin{cases} x & x\geq0,\\ -x+2& x< 0, \end{cases}\displaystyle \qquad B(x)= \textstyle\begin{cases} \frac{5}{4} & x\geq1,\\ \frac{1}{4} & x< 1, \end{cases}\displaystyle \\ C(x)= \textstyle\begin{cases} x & x\geq1,\\ 0 & x< 1, \end{cases}\displaystyle \qquad D(x)= \textstyle\begin{cases} -4x+1 & x\geq0,\\ x& x< 0. \end{cases}\displaystyle \end{gathered} $$

Obviously, “$\preceq_{i}$” is *d*-regular, $i = 1,2$. Moreover, *A* and *B* are comparable continuous mappings. If for some $x\in X$, we have $Ax\preceq Bx$, then $x\in[0,\frac{1}{4}]\cup[1,\frac{5}{4}]$ which implies that $Tx \in[0,\frac{1}{8}]$. Therefore

$$C(Tx)=0\leq-4Tx+1=DTx. $$

Thus *T* is $(A,B,C,D,\preceq_{1},\preceq_{2})$-stable. If for some $x\in X$, we have $Cx\leq Dx$ then $x\in[0,\frac{1}{4}]$, which implies that $Tx\in[0,\frac{1}{8}]$. Therefore

$$ATx=Tx\leq\frac{1}{4}= BTx. $$

Thus *T* is $(C,D,A,B,\preceq_{2},\preceq_{1})$-stable. Define $h:X\times X\rightarrow[0,1)$ and $\alpha:X\times X\rightarrow \mathbb{R}$ as follows:

$$ \alpha(x,y)= \textstyle\begin{cases} 1& [x]=[y],\\ 0&\text{otherwise}, \end{cases}\displaystyle \quad \text{and} \quad h(x,y)= \frac{1}{2}. $$

If $Ax\leq Bx$, $Cy\leq Dy$ and $\alpha(x,y)= 1$, then $x,y\in[0,\frac {1}{4}]$. Therefore

$$\begin{aligned} \alpha(x,y)d(Tx,Ty) =&\frac{1}{2}|x-y| \\ = & h(x,y)d(x,y) \\ \leq& h(x,y)M_{a}(x,y). \end{aligned}$$

Let $\phi(t)=t$, $t\geq0$. Therefore

$$ (Ax\preceq_{1} Bx\text{ and }Cy\preceq_{2} Dx) \quad \Longrightarrow\quad \alpha (x,y)\phi\bigl(d(Tx,Ty)\bigr)\leq h(x,y)\phi \bigl(M_{a}(x,y)\bigr). $$

The hypotheses of Theorem 2.1 are satisfied. Therefore (1.1) has the unique solution $x^{*}=0$.

Note that the mappings *A*, *B* and *T* are not continuous and $(X,d)$ is not a complete metric space.

## Consequences

Now, we consider some special cases, where in our result we deduce several well-known fixed point theorems of the existing literature.

### Corollary 3.1

([6])

*Let* $(X,d)$ *be a complete metric space endowed with two partial orders* ⪯_1_ *and* ⪯_2_. *Let* $T,A,B,C,D:X\rightarrow X$ *be given operators*. *Suppose that the following conditions are satisfied*:

(i) $\preceq_{i}$ *is d*-*regular*, $i = 1,2$;
(ii) *A* *and* *B* *are continuous*;
(iii) *there exists* $x_{0} \in X$ *such that* $Ax_{0} \preceq_{1} Bx_{0}$;
iv *T* *is* $(A,B,C,D,\preceq_{1},\preceq_{2})$-*stable*;
(v) *T* *is* $(C,D,A,B,\preceq_{2},\preceq_{1})$-*stable*;
(vi) *there exists* $\psi\in\Psi$ *such that*
  3.1 $$ Ax \preceq_{1} Bx, \qquad Cy \preceq_{2} Dy\quad \Rightarrow\quad d(Tx,Ty)\leq d(x,y)-\psi\bigl(d(x,y)\bigr). $$

*Then the sequence* $\{T^{n}x_{0}\}$ *converges to some* $x^{*}\in X$, *which is a solution to* (1.1). *Moreover*, $x^{*}$ *is the unique solution to* (1.1).

### Proof

Define $h:X\times X\rightarrow[0,1)$, by

3.2 $$ h(x,y)= \textstyle\begin{cases} \frac{d(x,y)-\psi(d(x,y)}{d(x,y)} & \mbox{if } x\neq y,\\ 0& \mbox{if }x=y. \end{cases} $$

Let $\{x_{n}\},\{y_{n}\}\subseteq X$ be such that sequence $\{d(x_{n},y_{n})\}$ is decreasing and $\lim_{n\rightarrow\infty} d(x_{n},y_{n})=r$. Suppose that $\lim_{n\rightarrow\infty}h(x_{n},y_{n})=1$. We show that $\lim_{n\rightarrow\infty} d(x_{n},y_{n})=0$. In the contrary case, let $\lim_{n\rightarrow\infty} d(x_{n},y_{n})=r>0$. Since *ψ* is lower semi-continuous,

$$\limsup_{n\rightarrow\infty} h(x_{n},y_{n})=\limsup _{n\rightarrow\infty} \frac{d(x_{n},y_{n})-\psi(d(x_{n},y_{n})}{d(x_{n},y_{n})}=\frac{r-\psi(r)}{r}=1, $$

which implies that $\psi(r)=0$, and so $r=0$. This is a contradiction. Therefore

$$\lim_{n\rightarrow\infty} d(x_{n},y_{n})=0. $$

This implies that $h\in\mathcal{H}(X)$. Let, for some $x,y\in X$, $Ax \preceq_{1} Bx$, $Cy \preceq_{2} Dy$. Then applying (3.1) and (3.2) we conclude that

$$d(Tx,Ty)\leq h(x,y)d(x,y)\leq h(x,y)M_{a}(x,y). $$

Also for all $x,y\in X$ define $\alpha(x,y)=1$. The hypotheses of Theorem 2.1 are satisfied. Hence there exists a unique $x^{*}\in X$ such that $x^{*}$ is the unique solution to (1.1). □

In Theorem 2.1, by setting ${\preceq_{1}}={\preceq_{2}}$, $C=B$ and $D=A$, we get the following corollary.

### Corollary 3.2

*Let* $(X,\preceq,d)$ *be a comparable complete metric space*(*not necessarily complete*) *with partial order* ⪯_1_. *Also*, *let operators* $T,A,B:X\rightarrow X$ *be given*. *Suppose that the following conditions are satisfied*:

(i) ⪯_1_ *is* *d*-*regular and* *T* *is* ⪯-*preserving and triangular* *α*-*admissible*;
(ii) *A*, *B* *and* *T* *are comparable continuous*;
(iii) *there exists* $x_{0} \in\mathcal{J_{T}}$ *such that* $Ax_{0} \preceq_{1} Bx_{0}$ *and* $\alpha(x_{0},Tx_{0})\geq1$;
(iv) *for all* $x\in X$, *we have*
  $$Ax\preceq_{1} Bx\quad \Longrightarrow\quad BTx\preceq_{1} ATx; $$
(v) *for all* $x\in X$, *we have*
  $$Bx\preceq_{1} Ax\quad \Longrightarrow\quad ATx\preceq_{1} BTx; $$
(vi) *there exist* $h\in\mathcal{H}(X)$ *and* $\phi\in\Phi$ *such that*
  $$ (Ax\preceq_{1} Bx\textit{ and }By\preceq_{1} Ay)\quad \Longrightarrow\quad \alpha (x,y)\phi\bigl(d(Tx,Ty)\bigr)\leq h(x,y)\phi \bigl(M_{a}(x,y)\bigr). $$

*Then the sequence* $\{T^{n}x_{0}\}$ *converges to some* $x^{*}\in X$ *satisfying* $Tx^{*}=x^{*}$ *and* $Ax^{*}=Bx^{*}$.

By setting $A=D=I_{x}$ and $C=B$ we have the following common fixed point theorem.

### Corollary 3.3

*Let* $(X,\preceq,d)$ *be a comparable complete metric space*(*not necessarily complete*) *with partial order* ⪯_1_. *Also*, *let operators* $T,A,B:X\rightarrow X$ *be given*. *Suppose that the following conditions are satisfied*:

(i) ⪯_1_ *is* *d*-*regular and* *T* *is* ⪯-*preserving and triangular* *α*-*admissible*;
(ii) *B* *and* *T* *are comparable continuous*;
(iii) *there exists* $x_{0} \in\mathcal{J_{T}}$ *such that* $x_{0} \preceq _{1} Bx_{0}$ *and* $\alpha(x_{0},Tx_{0})\geq1$;
(iv) *for all* $x\in X$, *we have*
  $$x\preceq_{1} Bx \quad \Longrightarrow\quad BTx\preceq_{1} Tx; $$
(v) *for all* $x\in X$, *we have*
  $$Bx\preceq_{1} x \quad \Longrightarrow\quad Tx\preceq_{1} BTx; $$
(vi) *there exist* $h\in\mathcal{H}(X)$ *and* $\phi\in\Phi$ *such that*
  $$ (x\preceq_{1} Bx \textit{ and } By\preceq_{1} y) \quad \Longrightarrow\quad \alpha (x,y)\phi\bigl(d(Tx,Ty)\bigr)\leq h(x,y)\phi \bigl(M_{a}(x,y)\bigr). $$

*Then the sequence* $\{T^{n}x_{0}\}$ *converges to some* $x^{*}\in X$ *satisfying* $Tx^{*}=x^{*}$ *and* $Bx^{*}=x^{*}$.

## Conclusions

In this note, we replace the completeness assumption of the space *X* with a weaker condition by introducing the concept of comparable complete metric spaces. So, we address a fixed point in the setting of incomplete metric spaces by using the constraint inequalities.
